# Enhancement of Solubility and Specific Activity of a Cu/Zn Superoxide Dismutase by Co-expression with a Copper Chaperone in *Escherichia coli*

**DOI:** 10.15171/ijb.1465

**Published:** 2016-12

**Authors:** Warawan Eiamphungporn, Sakda Yainoy, Virapong Prachayasittikul

**Affiliations:** ^1^Department of Clinical Microbiology and Applied Technology, Faculty of Medical Technology, Mahidol University, Bangkok 10700, Thailand; ^2^Center for Research and Innovation, Faculty of Medical Technology, Mahidol University, Bangkok 10700, Thailand

**Keywords:** Cu/Zn superoxide dismutase, Co-expression, Human copper chaperone

## Abstract

**Background:**

Human Cu/Zn superoxide dismutase (hSOD1) is an antioxidant enzyme with potential as a therapeutic agent. However, heterologous expression of hSOD1 has remained an issue due to Cu^2+^ insufficiency at protein active site, leading to low solubility and enzymatic activity.

**Objectives:**

The effect of co-expressed human copper chaperone (hCCS) to enhance the solubility and enzymatic activity of hSOD1 in *E. coli* was investigated in the presence and absence of Cu^2+^.

**Materials and Methods:**

*pETDuet-1-hSOD1* and *pETDuet-1-hCCS-hSOD1* were constructed and individually transformed into *E. coli* strain BL21(DE3). The recombinant hSOD1 was expressed and purified using immobilized metal affinity chromatography. The yield and specific activity of hSOD1 in all conditions were studied.

**Results:**

Co-expression with hCCS increased hSOD1 solubility at 37°C, but this effect was not observed at 25°C. Notably, the specific activity of hSOD1 was enhanced by 1.5 fold and greater than 3 fold when co-expressed with hCCS at 25°C with and without Cu^2+^ supplement, respectively. However, the chaperone co-expression did not significantly increase the yield of hSOD1 comparable to the expression of hSOD1 alone.

**Conclusions:**

This study is the first report demonstrating a potential use of hCCS for heterologous production of hSOD1 with high enzymatic activity.

## 1. Background

Superoxide dismutase (SOD) is a primary defense that acts to catalytically remove superoxide anions. In mammals, three forms of SOD have been distinguished by metal cofactors and localization (1). Amongst which, SOD1 or Cu/Zn SOD is the one of crucial enzymes, and typically the most abundant one (2,3). SOD1 is a homodimer consisting of two ~16 kDa subunits found in the cytoplasm and nucleus of the cell. It is a metalloenzyme, which its active sites contain two copper and one zinc ions per molecule (4). The copper ions are required for enzymatic activity, whereas the zinc ion only helps to stabilize the enzyme structure (5). Cu/Zn SOD is considered as a therapeutic agent for diseases mediated by oxidative stress (6-8). It has been reported that SOD1 could reduce inflammation (9), protect against reperfusion damage of ischemic tissue (10), and prevent oncogenesis (11). Efficient procedures for SOD1 production are important for clinical applications, therefore, the simple expression and purification procedures with high specific activity are of interest. Heterologous expression of hSOD1 has been conducted in many expression systems including *E. coli* (12,13), yeast (14,15), insect (16,17) and plant cells (18). However, the most common problem has been that the produced protein is Cu^2+^-deficient at active site resulting in low solubility and enzyme activity (12-14). The metal reconstitution *in vitro* is a method to incorporate Cu^2+^ into the apoenzyme, but it requires low pH that is harmful and consequently results in large losses of protein (19). Although addition of Cu^2+^ into the *E. coli* culture was reported to improve the Cu^2+^ incorporation, the production of Cu/Zn SOD with a full Cu^2+^ complement was still a complication. This could be due to a lack of Cu^2+^ delivery system in *E. coli*.

In eukaryotes, Cu^2+^ incorporation into the SOD1 *in vivo* is mediated by the action of the copper metallochaperone (copper chaperone for SOD1 or CCS). Studies with the yeast metallochaperone (yCCS) have shown that yCCS directly incorporates copper into SOD1 despite exquisitely low levels of available free copper (20). Moreover, co-expression of yCCS with human Cu/Zn SOD variants and pseudo-EC-SOD enhanced the protein yields with high copper content in the presence of Cu^2+^ supplement (19). SOD1 is activated principally via a CCS and to a lesser degree by a CCS-independent pathway of unknown mechanism in mammals (21). However, the effect of co-expression of hCCS on hSOD1 production in *E. coli* has never been elucidated.

## 2. Objectives

In this study, the co-expression of hCCS and hSOD1 in *E. coli* was accomplished in the optimized condition to gain higher SOD1 solubility and specific activity.

## 3. Materials and Methods

### 3.1.Construction of Plasmids for Co-expression of hSOD1 and hCCS

*E. coli* strain NovaBlue (Novagen, Germany) was used for cloning. *hSOD1* (GenBank: EF151142.1) was amplified from the pET20b-*hSOD1*, a construct donated by Prof. Daret K. St. Clair, University of Kentucky, using i-*Taq* polymerase (Intron Biotechnology, South Korea) with forward (5´- ATACATATGGCGACGAAGGC- 3´, underlined is *Nde*I restriction site) and reverse (5´-ATTGCTCAGCTTATTGGGCG-3´, underlined is *Bpu*1102 I). *hCCS* (GenBank: NM_005125) was amplified using Gene PoolTM cDNA, from human normal brain tissue (Invitrogen, USA) with forward (5´-TGGCCATGGCTTCGGATTCG- 3´, underlined is *Nco*I restriction site) and reverse (5´- GACAAGCTTTCAAAGGTGGG- 3´, underlined is *Hind*III restriction site). The 465 bp and 824 bp PCR products of *hSOD1* and *hCCS*, respectively were digested with the corresponding restriction enzymes and purified from agarose gel after electrophoresis. The *hSOD1* was ligated into plasmid *pETDuet-1* (Novagen, Germany) at multiple cloning site2 (MCS2) to obtain *pETDuet-1-hSOD1*. The *hCCS* was subsequently ligated into *pETDuet-1-hSOD1* at multiple cloning site1 (MCS1) to obtain *pETDuet-1- hCCS-hSOD1*. The recombinant plasmids were verified by DNA sequencing.

### 3.2. Co-expression of hSOD1 and hCCS

The protein expression was carried out in *E. coli* BL21(DE3) (Novagen, Germany). The transformed strains harboring recombinant plasmids were inoculated and grew for 16 h at 37ºC in LB containing 100 μg.mL^-1^ ampicillin. The culture was diluted into 3 L terrific broth (TB) containing 100 μg.mL^-1^ ampicillin and incubated at 37°C, 150 rpm until OD_600_ of 0.5. The target proteins were induced by addition of isopropyl- β-D-thiogalactopyranoside (IPTG) (Bio Basic Canada Inc. Canada) at a final concentration of 1 mM with/without 50 ppm CuCl_2_ (Bio Basic Canada Inc. Canada). The cultures were incubated at 25ºC for an additional 16 h. Cells were harvested by centrifugation (20,000 ×g, 20 min) and suspended in buffer A (50 mM phosphate buffer, pH 7.4) followed by sonication. The lysates were cleared by centrifugation at 20,000 ×g for 20 min.

### 3.3. Purification of hSOD1

The clear lysates were incubated for 30 min at 60°C to precipitate contaminating proteins. Cu/Zn SOD proteins are thermostable proteins, whereas most proteins precipitate at high temperature (19,22). The supernatant was filtered and loaded on a Ni-NTA sepharose column which pre-equilibrated with buffer A (50 mM phosphate buffer, pH 7.4) with ÄKTA prime protein purification system (GE healthcare life sciences, UK). After elution with a linear gradient of buffer B (buffer A + 1 M imidazole), the target protein containing fractions were combined. The imidazole was removed and purified proteins were concentrated by an Amicon Ultra 10,000-MWCO filter (Millipore Corp., USA). Protein molecular weight and purity under denaturing condition were determined by SDS-PAGE. Protein concentrations were measured by Bradford method (23), before storage at -80°C.

### 3.4. Enzymatic Activity and Spectral Property of hSOD1

SOD activity was measured according to the inhibition of nitroblue tetrazolium (NBT) reduction by superoxide radicals generating from NADH/phenazine methosulfate (PMS) reaction at aerobic and non-acidic pH conditions as previously described (24). The absorbance at 560 nm was monitored during 5 min as an index of NBT reduction using a UV-visible spectrophotometer and calculated the enzyme inhibition (%) to define the half maximal inhibitory concentration values (IC50) and specific enzymatic activity. One unit of SOD activity is defined as the amount of enzyme that causes 50% decrease in NBT reduction.

The absorption spectra in the visible region (500-800 nm) of ~1 mM protein solutions were obtained using a UV-2450 UV-visible spectrophotometer (Shimadzu, Japan).

### 3.5. Statistical Analysis

Data are presented as mean±standard deviation (SD). Comparison of two means was performed using paired t-test, p-value < 0.05 with 2-tailed t-test. All statistical calculations were performed using PASW statistic 18 (SPSS Inc., USA).

## 4. Results

In this study, the co-expression of hCCS and hSOD1 in *E. coli* was accomplished in the optimized condition to gain higher SOD1 solubility and specific activity.

### 4.1. Construction and Co-expression of hSOD1 and hCCS

**Figure 1:**
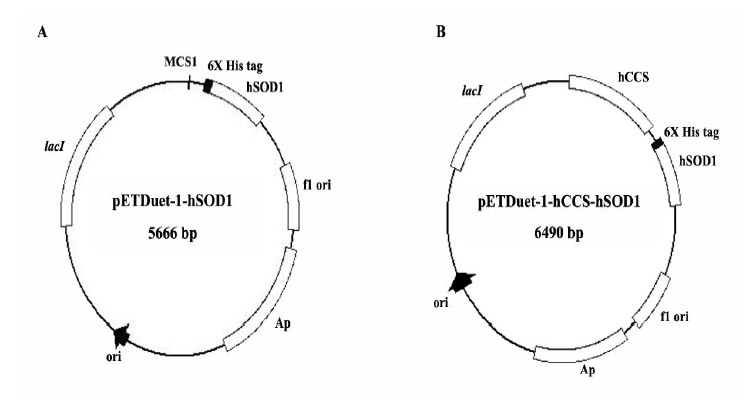
Construction of *pETDuet-1-hSOD1* and *pETDuet-hCCS-hSOD1*. A: The coding frame of *hSOD1* (465 bp) was cloned into MCS2. It is located downstream of 6× His tag. B: The coding frame of *hCCS* (824 bp) was cloned into MCS1

In this study, *pETDuet-1-hSOD1* and *pETDuet-1-hCCS-hSOD1* were successfully constructed (Figure 1).

**Figure 2:**
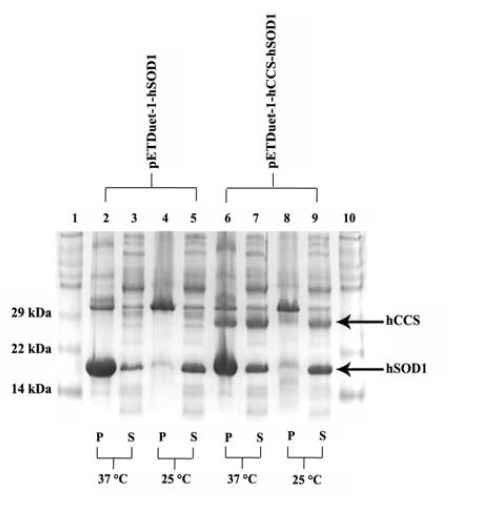
hSOD1 heterologous expression through the *pETDuet-1* expression system. SDS-PAGE showing induction of hSOD1 expression at 37ºC and 25ºC for 16 h. Lane 1 and 10, protein marker; Lane 2-5, fractions from *E. coli* BL21(DE3) carrying *pETDuet-1-hSOD1*; Lane 6-9, fractions from *E. coli* BL21(DE3) carrying *pETDuet-1-hCCS-hSOD1*; P, insoluble fraction; S, soluble fraction. Arrows indicate expected hCCS and hSOD1 proteins, MW~29 kDa and ~16 kDa, respectively. Results are representative of two separate experiments

*pETDuet*-*1*, a bicistronic expression vector, contains two multiple cloning sites (MCS1 and MCS2) that each includes a T7 promoter/lac operator and a ribosome binding site. The *hCCS* was cloned into MCS1 without a tag, whereas the *hSOD1* was cloned into a MCS2 with a polyhistidine tag that applied for hSOD1 purification. The transformed *E. coli* BL21(DE3) strains were cultivated in the medium without CuCl2 supplement. hSOD1 was highly expressed as insoluble form when the expression of *pETDuet-1-hSOD1* and *pETDuet-1-hCCS-hSOD1* were induced at 37°C (Figure 2).

However, in the case of co-expression with hCCS, a significant improvement in hSOD1 solubility was observed (Figure 2, lanes 3 and 7). Notably, lowering of induction temperature to 25ºC showed an even more increase in the solubility of hSOD1 with no significant enhancement in solubility in the presence of hCCS (Figure 2, lanes 3, 5, 7 and 9).

### 4.2. Purification of hSOD1

**Figure 3:**
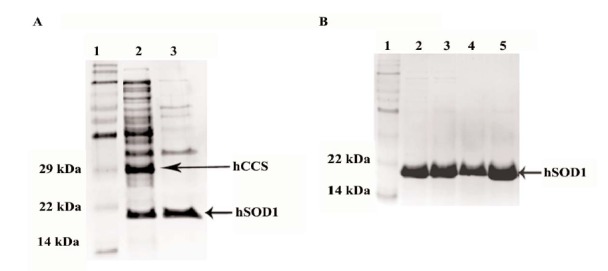
Protein expression and purification of recombinant hSOD1. A: Co-expression of hCCS at 25ºC (+CuCl2). Lane 1 protein marker; Lane 2, soluble fraction; Lane 3, soluble fraction after incubation at 60ºC for 30 min. B: Purification of recombinant hSOD1 using IMAC. Lane 1, protein marker; Lane 2, clone carrying *pETDuet-1-hSOD1* (-CuCl2); Lane 3, clone carrying *pETDuet-1-hSOD1* (+CuCl2); Lane 4 clone carrying pETDuet-1-hCCS-hSOD1 (-CuCl2); Lane 5 clone carrying pETDuet-1-hccs-hsoD1 (+CuCl2)

hSOD1 was further produced from *E. coli* under low temperature condition. Purification of His-tagged hSOD1 was conducted using immobilized metal affinity chromatography (IMAC). In the absence of hCCS co-expression, hSOD1 was purified to sufficient homogeneity by single step. However, in case of coexpression with hCCS, this copper chaperone could not be removed using only IMAC purification (data not shown). To solve this problem, protein supernatants obtained from *E. coli* lysates were incubated at 60°C for 30 min and centrifuged to remove the contaminating proteins, particularly hCCS (Figure 3A, lane 3). To examine whether the yields can be enhanced by co-expression of hCCS, *E. coli* carrying **pETDuet-1-hSOD1** and *pETDuet-1-hCCS-hSOD1* were grown in media in the presence or absence of 50 ppm CuCl2 supplement. All supernatants were applied to IMAC column. After purification procedure, the protein samples were resolved by 12% SDS-PAGE to confirm the hSOD1 purity. As shown in Figure 3B, hSOD1 was purified to homogeneity (> 95% purity) in all conditions. The final yields of purified hSOD1 as determined by Bradford were shown in Table 1.

**Table 1:** Effects of hCCS co-expression on yields of purified hSOD1

** Vectors **	** Supplementation of 50 ppm CuCl_2_**	** Yield of hSOD1 protein (mg.L^-1^ of culture) **
pETDuet-1-hSOD1 pETDuet-1-hSOD1 pETDuet-1-hCCShSOD1 pETDuet-1-hCCShSOD1	- + - +	20 31 22 30

### 4.3. Enzymatic Activity and Spectral Property of hSOD1

**Figure 4:**
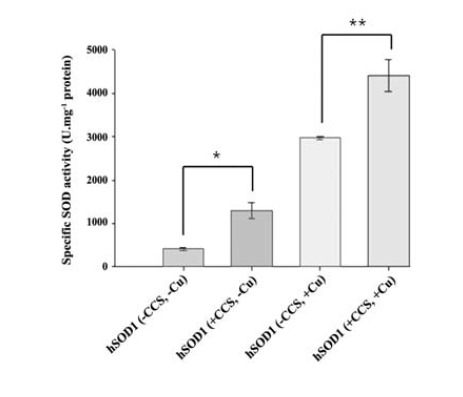
Specific activity of recombinant hSOD1 from clones with and without hCCS co-expression in the absence or presence of Cu^2+^ supplementation. One unit of SOD activity was defined as the amount of enzyme that caused 50% decrease in NBT reduction. Error bars represent the standard deviations of results obtained from three experiments in triplicates. The statistical analysis was evaluated by paired t-test. Values that are significantly different (P < 0.05) are indicated by asterisks

**Figure 5:**
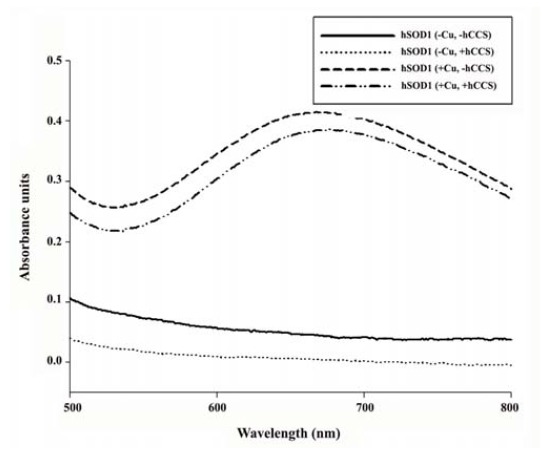
Visible absorption spectra of recombinant hSOD1. Spectra were observed and recorded from 1 mM hSOD1 in 50 mM phosphate buffer pH 7.4. The characteristic spectrum of Cu/Zn SOD is in the visible region with an absorption maximum at 680 nm. Solid line, purified hSOD1 (without hCCS, no Cu^2+^ supplement); dotted line, purified hSOD1 (no Cu^2+^ supplement, with hCCS); dash line, purified hSOD1 (Cu^2+^ supplement, without hCCS); dash-dotdot line, purified hSOD1 (Cu^2+^ supplement, with hCCS)

In eukaryotes, CCS has been known as the chaperone that directly incorporates Cu^2+^ into SOD1 *in vivo*. The presence of Cu^2+^ in the active site of Cu/Zn SOD enzymes is crucial for the activity of the enzyme. To investigate whether co-expression of hCCS enhances the enzymatic activity in *E. coli* expression system, the specific SOD activity was examined. Apparently, the result showed that co-expression of hCCS significantly increased the specific activity of hSOD1 in both the presence and absence of Cu^2+^ supplement. The highest activity was observed when supplementing with Cu^2+^ (Figure 4). The specific activity of hSOD1 produced by hCCS co-expression with Cu^2+^ supplement was approximately 1.5 fold greater than that of hSOD1 produced without co-expression (4,413±169 and 2,973±40 U.mg^-1^ protein, respectively). Interestingly, a 3-fold increase in the specific activity of hSOD1 was observed when hCCS was co-expressed as compared with no chaperone (1,298±187 and 414±29 U.mg^-1^ protein, respectively) in the absence of Cu^2+^ supplement. Since Cu/Zn SOD has a characteristic spectrum in the visible region with an absorption maximum at 680 nm (25), the visible absorption spectroscopy of the purified proteins was determined. Analysis of spectral property revealed the peak of hSOD1 at 680 nm obtained only from *E. coli* in Cu^2+^ supplemented medium (Figure 5). The spectrum was similar to previous study which representing the correct occupation of Cu (II) at active site (25). In contrast, no peak of hSOD1 was observed when *E. coli* was cultured in medium without Cu^2+^ supplement.

## 5. Discussion

hSOD1 is a metalloenzyme, which lack of Cu^2+^ at active site impairs its structure and maturation leading to low solubility and enzyme activity. Previous study indicated that hCCS is critical for maturation of hSOD1 through insertion of the Cu^2+^ and oxidization of an intra-subunit disulfide (26). Moreover, it has been demonstrated that hCCS, by interacting with the immature fALS (familial amylotrophic lateral sclerosis) SOD1 mutants, could exert a role of molecular chaperone for SOD1 both *in vivo* and *in vitro* (27). Herein, the effect of hCCS co-expression on hSOD1 solubility and enzymatic activity in *E. coli* expression system was firstly established using *pETDuet-1* expression vector. Our results represented that hCCS co-expression significantly increased the hSOD1 solubility at 37ºC but not at 25ºC in the presence of Cu^2+^ supplement. It is possible that the hCCS functioned at 37°C to reduce protein folding defects, whereas these defects were minimized and protein was folded properly at low temperature. Moreover, the co-expression with hCCS at 25°C did not significantly increase the yield of hSOD1 in both the absence and presence of Cu^2+^ supplement as compared to the expression of hSOD1 alone. This result indicated that the protein yield seemed to be affected by Cu^2+^ rather than hCCS. Interestingly, incubation of lysates at 60ºC during purification step did not affect the enzymatic activity of hSOD1. This result is consistent with the previous study that demonstrated the Cu/Zn SOD is a thermostable protein (22). Notably, our expression and purification systems produced higher amounts of recombinant hSOD1 (30 mg.L^-1^ of culture) when supplementing with Cu^2+^ as compared to previous study using *E. coli* (10 mg.L^-1^ of culture), yeast (7.6 mg.L^-1^), insect cells (~5-15 mg.L^-1^ of culture) and protozoa (6.5 mg.L^-1^ of culture) as expression hosts (13,15,17,28). Although, the different yields might be influenced by the different expression and purification systems used, the advantages of our system when compared to the eukaryotic expression systems are more rapid, simple and cost effective. Our results also demonstrated that co-expression with hCCS in the presence of Cu^2+^ supplement conferred higher enzymatic activity when compared with no chaperone. Moreover, the effect of hCCS on specific activity was clearly observed in the absence of Cu^2+^ supplement. This phenomenon suggested that hCCS actively functions in both Cu^2+^ abundant or depleted conditions, but it is more active in Cu^2+^ insufficient condition. This finding is in good agreement with the previous study that showed the better activity of yCCS under conditions where the free copper in the cytoplasm is strictly limited (20). Additionally, our results showed that supplementation of Cu^2+^ could increase the specific activity of hSOD1 in cells lacking of hCCS. This result is consistent with previous study that showed the activation of hSOD1 *in vivo* could be CCS-independent when copper concentrations were elevated in the growth medium (20). The specific activity of hSOD has been reported in range of 2,700-5,600 U.mg^-1^ protein, based on different expression hosts (13,15,29). Even though the highest activity was presented when hSOD1 was expressed and purified from transgenic rat tissue (~5,600 U.mg^-1^ protein), the expression yield was relatively low (29). Apparently, most expression systems demonstrated the lower specific activity of enzyme when compared to that of our system (~4,500 U.mg^-1^ protein). Interestingly, co-expression of hCCS did not affect the peak of purified hSOD1, although our results apparently showed that the specific activity of hSOD1 dramatically increased when the enzyme was coexpressed with hCCS chaperone. This result is inconsistent with previous study that displayed the coexpression with yCCS increased the metallization of hSOD-proteins with 87-98% copper saturation (19). However, our result is in good agreement with earlier study that showed the co-expression of CotA laccase with CopZ copper chaperone of *Bacillus licheniformis* in *E. coli* increased the specific activity of enzyme even though total copper content did not alter (30). Taken together, our results indicate that not only intracellular Cu^2+^ concentration, but also the presence of an appropriate copper chaperone affects the specific SOD activity. Our procedure is simply and can routinely be used for improved heterologous production of hSOD1 in *E.coli*.

## 6. Conclusions

This study firstly elucidates that the co-expression with hCCS in E. coli could increase the hSOD1 solubility and specific activity. Notably, hCCS co-expression affected the hSOD1 solubility when co-expressing at 37°C rather than 25°C. Moreover, the specific activity of hSOD1 was improved when co-expressing with hCCS at 25 °C in both presence and absence of supplementary Cu^2+^ but the highest activity was observed when supplementing with Cu^2+^. Interestingly, the effect of hCCS on hSOD1 specific activity was apparently showed in the absence of supplementary Cu^2+^. In addition, the chaperone coexpression did not significantly enhance the yield of hSOD1 comparable to the expression of hSOD1 alone. However, our expression and purification systems clearly demonstrated the high production of recombinant hSOD1. Taken together, the findings of this study present the application of hCCS in hSOD1 production for therapeutic use in the future.

## Acknowledgements

Authors would like to thank Prof. Daret K. St.Clair, University of Kentucky for providing *hSOD1*.

## Funding/Support

This work was supported by the Office of the Higher Education Commission and Mahidol University under the National Research Universities Initiative.
